# Rural–urban migration as a factor associated with physical and sexual intimate partner violence Peru 2015–2017: a secondary analysis of a national study

**DOI:** 10.1186/s12905-022-01648-7

**Published:** 2022-03-12

**Authors:** Jorge Terrazas, Dora Blitchtein

**Affiliations:** Universidad Peruana de Ciencias Aplicadas, Avenida Alameda San Marcos 11, Chorrillos, 15067 Lima Peru

**Keywords:** Intimate partner violence, Rural-to-urban migration, Demographic health survey migration

## Abstract

**Background:**

Internal migration, a consequence of the demographic transition towards urbanization driven by globalization, represents a particular public health challenge. Change in residence from one sociocultural geographic context to another, with not only economic implications, but also changes in women’s long-established relationships of family interdependence, influences gender relations and can influence Intimate Partner Violence (IPV) against women. Different migratory trajectories may be related to IPV. The aim of this study was to identify the association between internal migration and physical and/or sexual violence against women in the last 12 months.

**Methods:**

A secondary analytical cross-sectional analysis of the publicly accessible 2015–2017 Demographic and Family Health Survey (DHS) was performed. The outcome variable was reported physical and/or sexual violence inflicted by the partner (IPV) during the last 12 months. Exposure variable was internal migration, operationalized from three questions: current place of residence, principal place of residence before 12 years of age and number of years of residence in the current place. Migrants were classified as those who reported having lived for 5 years or more in the current location and were categorized as rural-rural migrants, urban-urban migrants, urban–rural migrants and rural–urban migrants, recent migrants and nonmigrants those who resided in the same place all their lives. To identify the association between internal migration and physical violence, a generalized linear model (GLM) of the family and the log Poisson link log option was used, and the results are presented as prevalence ratios (PRs). A crude model and a model adjusted for confounding variables were performed.

**Results:**

Rural–urban migrant women had a 15.0% higher probability of experiencing IPV than nonmigrant women (PRa 1.15, 95% CI 1.03–1.29, *p* = 0.015), while the probability of experiencing IPV in the last 12 months for urban–rural, rural-rural,urban-urban migrantand recent migrant women was not significantly different from that of nonmigrant women.

**Conclusion:**

Rural–urban migration among women of childbearing age is a factor associated with a higher probability of IPV in the last 12 months. The identification of women with this rural–urban migration pattern could help prioritize those that may experience a greater probability of physical and/or sexual violence in Peru, it must be studied if this pattern is the same in other countries.

## Background

Intimate partner violence, (IPV) against women is a topic of public health interest due to its presence in varying degrees in all societies and cultures [1]. It is estimated that approximately one in three women in the world experiences physical and/or sexual violence, with consequences for their quality of life, physical and/or mental health, the health of their children and other people in their environment and for society as a whole [2–4]. The complex nature of IPV is the result of multiple interacting factors, some of which are not yet clearly identified. Thus, it is necessary to address and understand IPV based on theories that consider the complexity of gender roles in various sociocultural contexts as well as the different levels at which this type of violence is manifested. IPV is conditioned by factors ranging from the individual level to the levels of interpersonal, institutional and social relationships. It is also the result of cultural and behavioral norms at all levels that influence the relationship [5–7].

In addition to the role of social dynamics, migration, a consequence of the demographic transition towards urbanization driven by globalization, represents a particular public health challenge that should be understood and included as a predictor of IPV [8, 9]. Internal migration, led mainly by women in most of the Latin American countries [10], involves a change in residence from one sociocultural geographic context to another and therefore influences gender relations and IPV against women; it also affects the people around them, the communities they settle in and their communities of origin. As such, it is necessary to explore different migration experiences and migratory trajectories as factors related to IPV [10–14].

In another context, a higher prevalence of IPV against women has been identified among migrants moving from rural to urban areas than among those who have not migrated, a finding that is explained in part by cultural and gender characteristics based on traditional and/or cultural social norms, low relationship satisfaction, extramarital sex and unstable living conditions [15].

In Peru, the IPV report for the last 12 months shows that although IPV has decreased in recent years, from 13.5% in 2009 to 10.6% in 2017, the change is not notable, and this figure is higher than in other parts of the world. However, within this change are differences according to indicators such as age, education, employment and wealth [16, 17].

The experiences of women in other contexts involving the migration from a rural to an urban area are related to poverty and the desire to improve their socioeconomic status, given the better job opportunities in urban areas. When gender relations are balanced during migration, both members of the couple engage in paid work, and the socioeconomic status of their families gradually improves with less likelihood of IPV. If this is not the case, and only the woman works, maintains the home and assumes domestic responsibilities, there is a reversal in gender relations that leads to an imbalance within the couple and a greater likelihood of IPV [11].

In Peru, as in other countries, internal migration involves adapting to different geographical areas and diverse sociocultural, economic and gender contexts [18–20]. Each member of the couple, according to his or her sociodemographic characteristics, health status, history and life experiences, will contribute differently to restricting the woman's autonomy and more likelihood of IPV against women. The different norms under which a relationship develops, which involve not only the couple but their friends and families, institutions and the roles assigned to men and women according to the social and cultural context, also predispose individuals towards IPV against women [21, 22]. It has not yet been revealed whether these characteristics of intimate partner violence in Peru are associated with some or all patterns of internal migration. The present study aims to identify the association between internal migration and physical and/or sexual violence against women in the last 12 months.

## Methods

For the present work, a secondary analytical cross-sectional analysis of the publicly accessible 2015–2017 Demographic and Family Health Survey (Encuesta Demográfica y de Salud Familiar, ENDES) was performed [23].

Records for women between 15 and 49 years of age who were randomly selected to respond to the domestic violence module of the ENDES individual survey for the period 2015–2017 and interviewed face-to-face by INEI personnel were included.

The ENDES used a two-stage probabilistic sample that was balanced, stratified and independent at the department level and analyzed by rural and urban areas. This type of sampling allows total estimates that are approximately equal to the characteristics of the reference population to be obtained [24].

The selection criteria were as follows. The inclusion criteria was women between 15 and 49 years who were selected to complete the violence module that included questions about physical and sexual violence and about where they lived for the longest until they were 12 years old, their current place of residence and the amount of time they lived there. The exclusion criterion was having lived abroad for most of their life before the age of 12 years [25].

### Measurement of variables

The outcome variable was reported physical and/or sexual violence inflicted by the partner (IPV) at some time during the last 12 months. This variable was developed based on ten questions regarding having ever being pushed, slapped, hit with a closed hand, kicked, suffering from an attempted strangling or burns and being threatened and/or attacked with a knife or firearm. Additionally, the participants were asked about being forced to have sexual relations and/or perform other sexual acts without consent. All questions had to be answered with “yes” or “no”, and if the answer was yes to any of these questions, the women were categorized as having reported IPV. The questions in the ENDES are a modified version of the Conflict Tactics Scale (CTS-2), which has high reliability and comparability among different cultures [26, 27].

The main exposure variable was internal migration. This was operationalized using information from three questions: current place of residence, principal place of residence before 12 years of age and number of years of residence in the current place. Migrants were classified as those who reported having lived for 5 years or more in the current location, this fixed interval of time lived is one of the internal migration recommended best measures [28–30] and were categorized as follows according to changes in their primary residence and current residence as rural-rural migrants, urban-urban migrants, urban–rural migrants and rural–urban migrants, recent migrants were those who reported having lived less than 5 years in the current location without migration pattern specification. Nonmigrants were those who reported having resided in the same place all their lives.

In addition, other variables were included, including age in years, educational level, marital status, native language, paid occupation of the interviewee, socioeconomic level, age at first marriage/cohabitation, age when sexual intercourse started, number of children, alcohol consumption by their stable partner in the last 12 months, number of sexual partners in addition to their stable partner in the last 12 months and history of physical aggression by their father toward their mother [15].

Statistical power was calculated using Open Epi, version 3.01, considering 7571 women who were exposed to IPV and 56,288 who were not exposed, as well as the prevalence of IPV in the last 12 months among migrant women from China (19.04%) [15] and the prevalence of IPV during the last 12 months in women in the general population of Peru (10.8%) [31]. With a confidence interval of 95%, the power was greater than 80%.

The present study was reviewed and approved by a Ethics Committee of the Universidad Peruana de Ciencias Aplicadas.

#### Data analysis

For the statistical analysis, the STATA 16 MP program was used, considering a 95% confidence level.

A descriptive analysis of the sociodemographic characteristics of the population and the prevalence of physical violence and internal migration was performed, and internal migration subgroups were identified. Simple frequencies and weighted percentages are reported for the categorical variables, and means and standard deviations are reported for the numerical variables.

Then, a bivariate analysis using the Pearson chi-square test was performed to identify the associations of the categorical variables. Numerical variables were compared by regression and the Wald test.

To identify the association between internal migration and physical violence, a generalized linear model (GLM) of the family and the log Poisson link option was used, and the results are presented as prevalence ratios (PRs). A crude model and a model adjusted for confounding variables were performed; confounding variables were entered into the model according to epidemiological criteria. To evaluate the collinearity between the independent variables that were included in the final adjusted model, the variance inflation factor (VIF) test was used, and a value of ten was used to exclude a variable from the model. Additionally, a correlation analysis of the variables was performed; a correlation greater than 0.5 was found between education level and socioeconomic level (0.052), so the latter was excluded from the final adjusted model. For the statistical analysis, the stratified design of the primary study was taken into account, and adjustments for sample weights were made using the "svy" commands included in STATA.

## Results

**Fig. 1:**
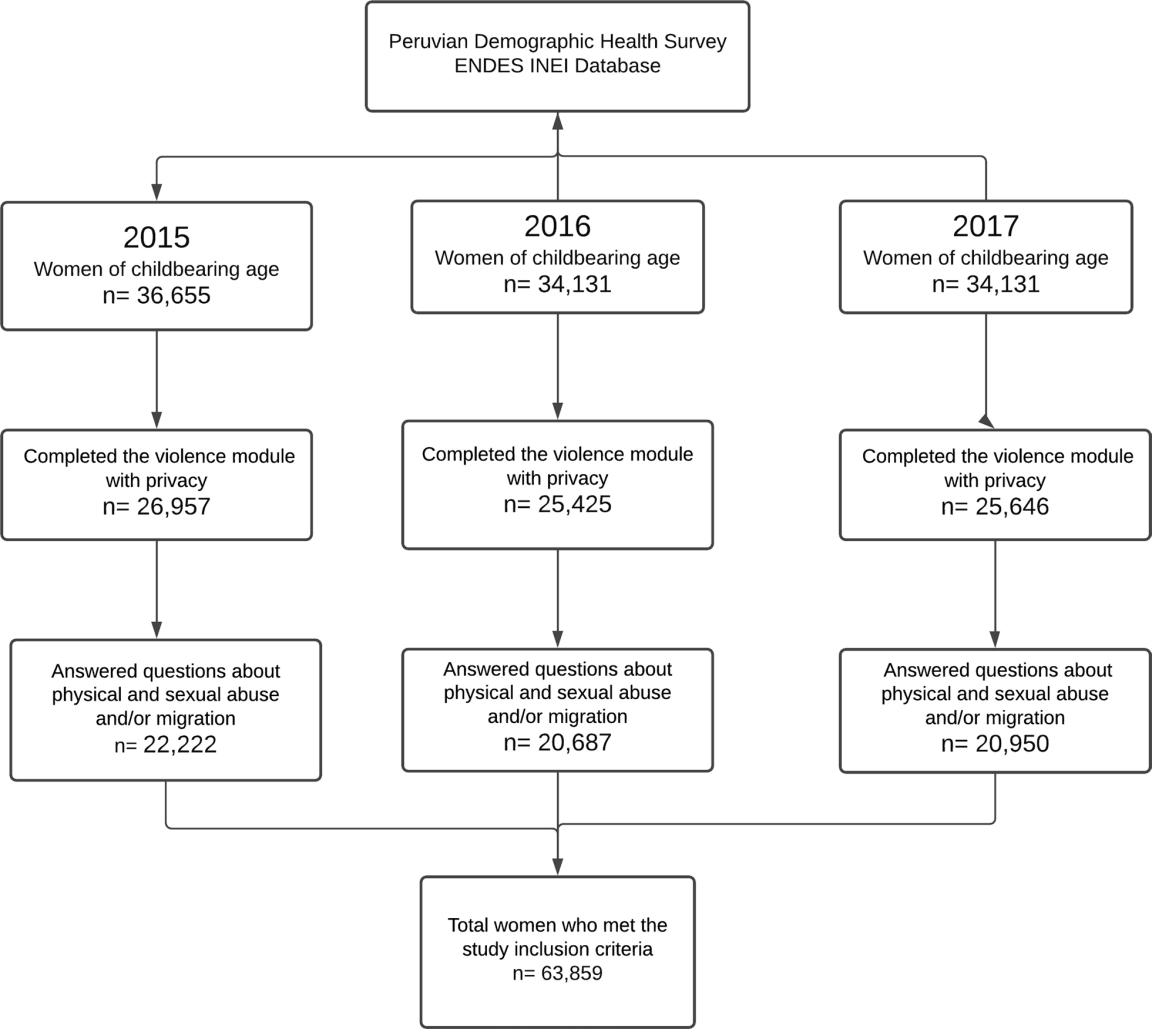
Women of child-bearing age who met the study selection criteria. Peru 2015–2017

The total number of women who met the selection criteria for this study was 63,859 (Fig. 1). Approximately one in ten women (11.0%) reported having experienced IPV in the last 12 months. Considering the factors related to violence at the individual level, around three in ten women (30.1%) were between 15 and 29 years old; 5.4% had a indigenous native Peruvian language (Quechua, Aymara or other) as their mother tongue; more than two in ten (25.4%) reported primary education or less as the highest level of education achieved; and 0.6% reported having had an STD diagnosis in the last 12 months (Table 1).

**Table 1:** Sociodemographic characteristics of women of childbearing age. Peru 2015–2017 (n = 63,859)

Levels	Sociodemographic Characteristics of women	n	(%)^a^	CI 95%	
				Ll^a^	Ul^a^
Individual	*Age*				
	15–29 years	24,956	(30.1)	29.5	30.8
	30–49 years	38,963	(69.9)	69.2	70.5
	*Education*^b^				
	None/Early education	1514	(2.1)	1.9	2.3
	Primary	16,049	(23.3)	22.4	24.2
	Secondary	28,442	(43.8)	42.8	44.8
	Higher (non-university)	10,521	(18.0)	17.3	18.6
	Higher (University and postgraduate)	7332	(12.9)	12.0	13.7
	*History of physical aggression by father toward mother*				
	No	33,842	(53.7)	52.9	54.5
	Yes	27,727	(42.9)	42.1	43.7
	Does not know/Did not answer	2290	(3.3)	3.1	3.6
	*Mother tongue*				
	Spanish	57,611	(93.1)	92.4	93.7
	Quechua	4959	(5.4)	4.9	6.0
	Aymara	346	(0.6)	0.4	0.9
	Other indigenous language/foreign language	943	(0.9)	0.7	1.2
	*Socioeconomic status*				
	Lower quintile	16,403	(19.2)	18.2	20.3
	Second quintile	17,381	(22.7)	21.6	23.7
	Third quintile	13,245	(21.4)	20.6	22.2
	Fourth quintile	9948	(19.7)	18.8	20.6
	Upper quintile	6882	(17.1)	15.9	18.4
	*Number of children*				
	None	1816	(6.0)	5.6	6.4
	One child	16,026	(24.9)	24.3	25.6
	Two children	19,937	(31.6)	30.9	32.2
	Three or more children	26,080	(37.5)	36.7	38.3
	*Interviewee has paid occupation*^c^				
	No	23,832	(33.6)	32.8	34.4
	Yes	40,024	(66.4)	65.6	67.2
	*STD reported in the last 12 months*				
	Does not know STD signs/simptoms	21,437	(29.6)	28.6	30.6
	No	41,983	(69.8)	68.8	70.8
	Yes	397	(0.6)	0.5	0.7
	Unknown	42	(0.1)	0.03	0.1
Relationships	*Marital status*				
	Married	18,116	(30.7)	29.8	31.5
	Cohabitating	38,093	(53.8)	52.9	54.6
	Separated/Divorced/Widowed	7650	(15.6)	15.0	16.2
	*More than one sexual partner in the last 12 months*^d^				
	No	60,675	(93.4)	93.0	93.8
	Yes	3170	(6.6)	6.2	7.0
	*Alcohol consumption by the partner in the last 12 months*^e^				
	Yes	12,537	(19.9)	19.2	20.5
	No	51,319	(89.2)	79.5	80.8
	*Health insurance*				
	No	26,402	(50.3)	49.1	51.4
	Yes	37,457	(49.7)	48.6	50.9
Social	*Internal migration*				
	Non-migrant	26,882	(45.4)	44.3	46.5
	Recent migrant	10,003	(12.0)	11.6	12.5
	Urban–urban	6548	(11.4)	10.8	12.0
	Urban–rural	1024	(1.1)	1.0	1.3
	Rural–urban	12,204	(21.3)	20.4	22.2
	Rural–rural	7198	(8.7)	8.2	9.3
	*Time living in the place to which you migrated*				
	Non-migrant	26,882	(45.4)	44.3	46.5
	Less than 5 years	10,003	(12.0)	11.6	12.5
	5 years or more	26,974	(42.6)	41.6	43.5

CI, confidence interval; IL, inferior limit; UL, upper limit

^a^Weighted percentages

^b^1 missing data

^c^3 missing data

^d^14 missing data

^e^3 missing data

Regarding internal migration, 45.4% were nonmigrants;12.0% were recent migrants, 21.3% were rural–urban migrants; 11.4% were urban-urban migrants; 8.7% were rural-rural migrants; and 1.1% were urban–rural migrants. Regarding the time spent at the current place of residence, 12.0% had resided there for less than 5 years; 42.6% had resided there for 5 years; or more; and the rest were nonmigrants (45.4%) (Table 1).

A total of 42.9% reported having witnessed physical aggression by their father toward their mother, and around 6.9% had a native language of Peru as their mother tongue (Quechua, Aymara or other) (Table 1).

Regarding their relationship with their partner, more than eight out of ten (84.5%) were married/cohabitating and 37.5% had more than two children; 6.6% reported having one or more than one sexual partner in addition to their stable partner in the last 12 months (Table 1).

Regarding community level factors, for every ten women, approximately four (41.9%) belonged to the two lowest socioeconomic quintiles; almost seven (66.4%) had a paid job, and five (50.3%) reported not having any health insurance (Table 1).

Concerning socioeconomic level and women internal migration, higher percentages of rural-rural migrants, urban-urban migrants, and recent migrants belonged to the two lowest socioeconomic quintiles than rural–urban migrants, non-migrants, and urban-urban migrants (95.2%, 82.1%, 59 0.4% vs. 35.8%, 34.7% and 18.2% *p* < 0.001). (Table 2) (Table 2).

**Table 2:** Association between internal migration and sociodemographic characteristics of women of childbearing age, Peru 2015–2017

	Internal migration																		
	Non-migrants			Recent-migrants			Urban–Urban			Urban–Rural			Rural–Urban			Rural–Rural			*P*^f^
Sociodemographic characteristics of migrant women	26,882 (45.4)	(CI 95% 44.3;46.5)		10,003 (12.0)	(CI 95% 11.5;12.5)		6,548 (11.4)	(CI 95% 10.8;11.9)		1,024 (1.1)	(CI 95% 1.0;1.3)		12,204 (21.3)	(CI 95% 20.4;22.2)		7,198 (8.7)	(CI 95% 8.2;9.3)		
		CI 95%			CI 95%			CI 95%			CI 95%			CI 95%			CI 95%		
	n (%^a^)	Ll^a^	Ul^a^	n (%^a^)	Ll^a^	Ul^a^	n (%^a^)	Ll^a^	Ul^a^	n (%^a^)	Ll^a^	Ul^a^	n (%^a^)	Ll^a^	Ul^a^	n (%^a^)	Ll^a^	Ul^a^	
*Age*																			
15–29 years	10,866 (31.0)	30.0	32.1	6,071 (54,9)	53.2	56.6	1,937 (21.6)	20.1	23.3	332 (28.2)	24.8	31.9	3,534 (20.5)	19.3	21.8	2,216 (25.9)	24.7	27.2	< 0.001
30–49 years	16,016 (68.9)	67.9	70.0	3,932 (45.1)	43.4	46.8	4,611 (78.4)	76.7	79.9	692 (71.8)	68.1	75.2	8,670 (79.5)	78.2	80.7	4,982 (74.1)	72.8	75.4	
*Education*^b^																			
None /Early education	494 (1.5)	1.3	1.8	207 (2.2)	1.8	2.7	29 (0.5)	0.3	0.9	28 (2.9)	1.8	4.4	243 (1.9)	1.5	2.4	513 (7.5)	6.6	8.4	< 0.001
Primary	5,232 (16.5)	15.5	17.8	2,545 (26.1)	24.6	27.6	557 (8.7)	7.5	10.3	305 (33.3)	29.1	37.8	3,377 (28.4)	26.9	30.1	4,033 (59.2)	57.3	61.0	
Secondary	12,037 (44.8)	43.4	46.3	4,836 (46.4)	44.8	48.1	2,863 (42.1)	40.0	44.6	497 (44.3)	39.9	48.9	5,879 (47.1)	45.4	48.7	2,330 (29.1)	27.3	30.8	
Higher (non-university)	5,254 (20.9)	19.9	21.9	1,423 (15.3)	14.1	16.7	1,600 (23.9)	22.1	25.9	131 (13.5)	10.8	16.8	1,862 (16.2)	15.0	17.5	251 (3.5)	2.8	4.3	
Higher (University and Postgraduate)	3,864 (16.2)	15.2	17.4	992 (10.0)	9.0	11.2	1,499 (24.7)	22.3	27.3	63 (5.9)	4.3	8.1	843 (6.3)	5.5	7.2	71 (0.9)	0.6	1.3	
*Socioeconomic status*																			
Lower quintile	6,536 (17.6)	16.4	19.0	3,201 (27.2)	25.7	28.9	177 (1.8)	1.4	2.3	468 (44.7)	39.3	50.3	994 (5.7)	5.0	6.6	5,027 (68.8)	65.9	71.5	< 0.001
Second quintile	5,837 (17.1)	16.1	18.2	3,389 (32.2)	30.4	34.0	1,410 (16.4)	14.4	18.5	398 (37.4)	33.2	41.7	4,488 (30.1)	28	32.2	1,859 (26.4)	24	28.7	
Third quintile	5,406 (19.7)	18.7	20.7	1,794 (20.2)	18.8	21.8	1,858 (25.0)	23.0	27.2	108 (11.8)	9.0	15.3	3,836 (31.6)	30	33.4	243 (3.6)	2.9	4.5	
Fourth quintile	5,119 (23.3)	22.0	24.6	1,030 (11.7)	10.5	13.0	1,707 (26.0)	24.0	28.1	31 (4.4)	2.4	7.9	2,005 (21.4)	20	23.2	56 (1.1)	0.7	1.6	
Upper quintile	3,984 (22.3)	20.7	24.0	589 (8.7)	7.5	10.2	1,396 (30.8)	27.8	34	19 (1.7)	0.9	3.3	881 (11.1)	9.7	12.6	13 (0.2)	0.1	0.4	
*Marital/conjugal status of the interviewee*																			
Married	8,151 (32.9)	31.6	34.2	1,884 (20.5)	19.1	21.9	2,041 (33.5)	31.3	35.9	255 (28.7)	24.5	33.2	3,360 (28.6)	27.0	30.2	2,425 (34.9)	33	36.8	< 0.001
Cohabitating	14.788 (48.6)	47.4	49.9	7,149 (67.9)	66.2	69.4	3,708 (50.6)	48.2	53.0	684 (60.8)	56.0	65.4	7,475 (56.5)	55	58.2	4,289 (57.8)	56	59.6	
Separated/ Divorced/Widowed	3,943 (18.5)	17.6	19.5	970 (11.7)	10.6	12.9	799 (15.9)	14.2	17.8	85 (10.6)	7.9	14.0	1,369 (15.0)	14	16.3	484 (7.4)	6.6	8.3	
*History of physical aggression by father toward mother*																			
No	15,161 (57.0)	55.8	58.1	5,249 (53.1)	51.3	54.9	3,301 (52.4)	50.2	54.6	463 (44.3)	40.1	48.6	5,868 (48.3)	47	50.0	3,800 (53.9)	52.0	55.7	< 0.001
Yes	10,855 (40.2)	39.1	41.4	4,411 (43.4)	41.7	45.1	3,059 (45.2)	43.1	47.4	497 (49.7)	45.3	54.2	5,884 (47.9)	46.2	49.5	3,021 (40.6)	38.8	42.5	
Does not know/Did not answer	866 (2.8)	2.5	3.1	343 (4.1)	2.9	4.1	188 (2.4)	1.8	3.2	64 (6.0)	4.3	8.3	452 (3.8)	3.2	4.5	377 (5.5)	4.8	6.4	
*Mother tongue*																			
Spanish	23,957 (93.2)	92.3	93.9	8,996 (91.4)	90.3	92.4	6,492 (99.1)	98.4	99.4	924 (90.0)	86.8	92.4	11,719 (97.0)	96	97.5	5,523 (78.3)	75	80.9	< 0.001
Quechua	2,175 (5.1)	4.5	5.8	810 (6.8)	5.9	7.8	41 (0.6)	0.3	1.0	90 (8.3)	6.2	11.0	401 (2.5)	2.1	3.0	1,442 (18.3)	15.8	21.0	
Aymara	138 (0.5)	0.3	0.9	66 (0.7)	0.5	1.1	9 (0.1)	0.1	0.3	4 (1.1)	0.2	4.4	44 (0.3)	0.2	4.4	85 (1.9)	1.1	3.2	
Other indigenous language/foreign language	612 (1.2)	0.8	1.7	131 (1.1)	0.7	1.6	6 (0.3)	0.1	1.1	6 (0.7)	0.3	1.8	40 (0.2)	0.3	1.8	148 (1.6)	1.1	2.5	
*Number of children*																			
None	818 (6.5)	5.8	7.2	544 (12.4)	11.0	14.0	161 (4.9)	3.8	6.1	17 (2.6)	1.4	4.8	195 (3.8)	3.0	4.8	81 (1.9)	1.5	2.4	< 0.001
One child	7,435 (27.2)	26.2	28.2	3,737 (32.9)	31.4	34.4	1,583 (25.9)	23.8	28.0	150 (15.0)	12.1	18.4	2,325 (21.0)	12	18.4	796 (12.0)	11	13.2	
Two children	8,705 (33.3)	32.2	34.3	2,895 (26.8)	25.4	28.2	2,283 (35.0)	33.4	37.0	315 (29.4)	25.6	33.4	3,925 (31.8)	30	33.3	1,814 (24.6)	23	26.0	
Three or more children	9,924 (33.1)	32.3	34.1	2,827 (27.9)	26.4	29.5	2,521 (34.3)	32.1	36.6	542 (53.1)	48.5	57.6	5,759 (43.4)	42	45.1	4,507 (61.6)	60	63.3	
*Interviewee has paid occupation*^c^																			
No	9,697 (32.6)	31.5	33.8	4,516 (44.2)	42.4	46.0	2,260 (29.5)	27.6	31.5	357 (32.6)	28.6	36.8	4,656 (32.7)	31	34.3	2,348 (31.7)	30	33.7	< 0.001
Yes	17,185 (67.4)	66.2	69.0	5,486 (55.8)	54.0	57.7	4,288 (70.5)	68.5	72.4	667 (67.4)	63.2	71.4	7,548 (67.3)	66	68.8	4,850 (68.3)	66	70.2	
*STD reported in the last 12 months*																			
Does not know STD signs/simptoms	8,161 (25.3)	24.0	26.6	3,849 (37.7)	35.9	40.0	1,088 (15.5)	13.9	17.2	347 (35.2)	30.7	40.0	3,891 (29.4)	28	31.0	4,101 (58.8)	57	61.1	< 0.001
No	18,561 (74.1)	72.8	75.4	6,083 (61.7)	59.9	64.0	5,392 (83.9)	82.1	85.5	666 (64.0)	59.2	68.5	8,214 (69.9)	68	71.4	3,067 (40.8)	39	43.1	
Yes	145 (0.6)	0.4	0.8	65 (0.5)	0.3	0.7	65 (0.6)	0.4	0.9	11 (0.9)	0.5	1.6	84 (0.7)	0.5	0.9	27 (0.3)	0.2	0.5	
Unknown	15 (0.04)	0.02	0.1	3 (0.1)	0.02	0.2	3 (0.04)	0.0	0.2	0 (0.0)	0,0	0,0	15 (0.1)	0	0.2	3 (0.02)	0	0.1	
*Another sexual partner in the last 12 months in addition to the stable partner*^d^																			
No	25,150 (91.9)	91.2	92.5	9,601 (95.1)	94.3	95.9	6,170 (91.9)	90.4	93.2	1,000 (97.4)	95.6	98.5	11,664 (94.4)	94	95.2	7,090 (98.4)	98	98.7	< 0.001
Yes	1,725 (8.1)	7.5	8.8	398 (4.9)	4.1	5.7	378 (8.1)	6.8	9.6	24 (2.6)	1.5	4.4	539 (5.6)	4.8	6.5	106 (1.6)	1.3	2.1	
*Alcohol consumption by the partner in the last 12 months*^e^																			
No	4,871 (18.8)	17.9	19.8	2,078 (19.6)	18.3	20.9	1,250 (21.9)	20.0	23.9	190 (19.6)	16.3	23.3	2,505 (20.3)	19.0	21.7	1,643 (21.9)	21	23.4	0.001
Yes	22,010 (81.2)	80.2	82.1	7,925 (80.4)	79.1	81.7	5,298 (78.1)	76.1	80.1	834 (80.4)	76.7	83.7	9,697 (79.7)	78	81.0	5,555 (78.1)	77	79.5	

CI, confidence interval; IL, inferior limit; UL, upper limit

^a^All percentages are weighted

^b^1 missing data

^c^3 missing data

^d^14 missing data

^e^3 missing data

^f^Pearson's chi2 with Rao Scott correction

Regarding the association between IPV in the last 12 months and the sociodemographic characteristics of women of childbearing age, a higher proportion of IPV was observed among who reported a history of physical aggression by their father towards their mother than among those who did not report it (14.5% vs. 8.1%, respectively; *p* < 0.001). In addition, reports of IPV were higher among women with a paid occupation than among those who were not employed (11.6% vs. 9.6%, respectively; *p* < 0.001). On the other hand, the number of women with IPV in the last 12 months who reported alcohol consumption by their partner in the last 12 months was higher than the number who did not (12.0% vs. 6.9%, respectively; *p* < 0.001) (Table 3).

**Table 3:** Association between physical intimate partner violence in the last 12 months and sociodemographic characteristics of women of childbearing age. Peru 2015–2017 (n = 63,859)

Levels		Report of violence in the last 12 months								*p*^h^
	Sociodemographic characteristics of women	Yes, violence reported				No reported violence				
		n = 7571 (11.0%)		(CI 95% 10.6; 11.5)		n = 56,288 (89.0%)		(CI 95% 88.5; 89.5)		
		n	(%)^b^	CI 95%		n	(%)^b^	CI 95%		
				Ll^b^	Ul^b^			Ll^b^	Ul^b^	
Individual	*Age*									
	15–29 years	3487	(13.6)	12.9	14.4	21,459	(86.4)	85.6	87.1	
	30–49 years	4074	(9.9)	9.4	10.4	34,829	(90.1)	89.6	90.7	
	*Education*^c^									
	None/Early education	163	(9.8)	8.1	11.9	1351	(90.2)	88.2	91.9	< 0.001
	Primary	1891	(11.9)	10.9	12.9	14,158	(88.2)	87.1	89.1	
	Secondary	3766	(12.5)	11.8	13.1	24,676	(87.6)	86.9	88.2	
	Higher (non-university)	1128	(9.1)	8.3	10.1	9393	(90.9)	89.9	91.7	
	Higher (University and Postgraduate)	622	(7.3)	6.4	8.4	6710	(92.7)	91.6	93.6	
	*History of physical aggression by father toward mother*									
	No	2962	(8.1)	7.6	8.7	30,880	(91.9)	91.3	42.4	< 0.001
	Yes	4331	(14.5)	13.8	15.3	23,396	(85.5)	84.7	86.2	
	Does not know/Did not answer	278	(11.5)	9.0	14.6	2012	(88.5)	85.4	91	
	*Mother tongue*									
	Spanish	6847	(10.9)	10.4	11.4	50,764	(89.1)	88.6	89.6	0.102
	Quechua	601	(12.6)	11.3	14.1	4358	(87.4)	85.9	88.7	
	Aymara	42	(15.1)	10.2	21.9	304	(85.0)	78.2	89.8	
	Other indigenous language/foreign language	81	(11.1)	7.0	11.5	862	(89.0)	83.0	93.0	
	*Socioeconomic status*									
	Lower quintile	1939	(11.3)	10.5	12.1	14,464	(88.7)	87.9	89.5	< 0.001
	Second quintile	2426	(13.6)	12.7	14.5	14,955	(86.4)	85.5	87.3	
	Third quintile	1696	(12.8)	11.8	13.9	11,549	(87.2)	86.1	88.2	
	Fourth quintile	1040	(10.2)	9.2	11.4	8908	(89.8)	88.7	90.8	
	Upper quintile	470	(5.9)	5.0	6.8	6412	(94.1)	93.2	95.0	
	*Number of children*									
	None	171	(8.5)	6.8	10.6	1645	(91.5)	89.4	93.2	< 0.001
	One child	1941	(11.3)	10.4	12.2	14,085	(88.7)	87.8	89.6	
	Two children	2260	(10.1)	9.4	10.9	17,677	(89.9)	89.1	90.6	
	Three or more children	3199	(12.0)	11.3	12.7	22,881	(88.0)	87.3	88.7	
	*Interviewee has paid occupation*^d^									
	No	2525	(9.8)	9.1	10.5	21,307	(90.2)	89.5	90.9	< 0.001
	Yes	5046	(11.6)	11.1	12.2	34,978	(88.4)	87.8	88.9	
	*STD reported in the last 12 months*									
	Does not know STD signs/simptoms	2499	(11.4)	10.7	12.3	18,938	(88.5)	87.7	89.3	< 0.001
	No	4961	(10.7)	10.2	11.2	37,022	(89.3)	88.8	89.8	
	Yes	105	(26.1)	19.2	34.6	292	(73.9)	65.5	80.9	
	Unknown	6	(8.9)	3.5	20.9	36	(91.1)	79.1	96.5	
Relationships	*Marital status*									
	Married	1485	(7.6)	6.9	8.3	16,631	(92.4)	91.7	93.1	< 0.001
	Cohabitating	4914	(12.3)	11.7	12.9	33,179	(87.7)	87.1	88.2	
	Separated/Divorced/Widowed	1172	(13.1)	11.9	14.5	6478	(86.9)	85.5	88.1	
	*More than one sexual partner in the last 12 months*^e^									
	No	7001	(10.8)	10.4	11.3	53,674	(89.2)	88.7	89.7	< 0.001
	Yes	565	(13.6)	11.8	15.5	2605	(86.4)	84.5	88.2	
	*Alcohol consumption by the partner in the last 12 months*^f^									
	Yes	875	(6.9)	6.1	7.8	11,662	(93.1)	19.2	20.5	< 0.001
	No	6696	(12.0)	11.5	12.5	44,623	(88.0)	87.5	88.5	
Social	*Internal migration*									
	Non-migrant	2950	(10.3)	9.7	10.9	23,932	(89.7)	89.1	90.3	< 0.001
	Recent migration	1344	(12.2)	11.3	13.3	8659	(87.8)	86.7	88.8	
	Urban–Urban	773	(9.8)	8.6	11.1	5775	(90.2)	88.9	91.4	
	Urban–Rural	151	(13.8)	11.1	16.9	873	(86.2)	83.1	88.9	
	Rural–Urban	1534	(12.5)	11.3	13.7	10,670	(87.6)	86.3	88.7	
	Rural–Rural	819	(10.8)	9.7	11.9	6379	(89.2)	88.1	90.3	
	*Time living in the place to which you migrated*									
	Non-migrant	2950	(10.3)	9.7	10.9	23,932	(89.7)	89.1	90.3	0.004
	Less than 5 years	1344	(12.2)	11.3	13.3	8659	(87.8)	86.7	88.8	
	5 years or more	3277	(11.4)	10.7	12.2	23,697	(88.6)	87.8	89.3	

CI, confidence interval; IL, inferior limit; UL, upper limit

^b^Weighted percentages

^c^1 missing data

^d^3 missing data

^e^14 missing data

^f^3 missing data

^h^Pearson's chi2 with Rao Scott correction

Regarding the association between internal migration and IPV in the last 12 months, the main result of this study, it was identified that, compared to nonmigrant women, urban–rural migrants had a 34.0% higher probability of experiencing IPV (PRc 1.34, 95% CI 1.07–1.66, *p* = 0.008), rural–urban migrants had a 21.0% higher probability of experiencing IPV (PRc 1.21, 95% CI 1.08–1.35, *p* = 0.001) and recent migrant had 19.0% higher probability of experiencing IPV (PRc 1.19, 95%.CI1.08 to1.31, *p* = 0.001). However, after adjusting for the confounding variables of age, educational level, having witnessed physical violence by the father toward the mother, native language, paid occupation, number of children, marital/conjugal status, having another sexual partner in addition to the stable partner in the last 12 months and alcohol consumption by the partner, rural–urban migrant women had a 15.0% higher probability of experiencing IPV than nonmigrant women (PRa 1.15, 95% CI 1.03–1.29, *p* = 0.015), while the probability of experiencing IPV in the last 12 months for urban–rural, rural-rural and urban-urban migrant women and recent migrant women was not significantly different from that of nonmigrant women (PRa 1.18, 95% CI 0.95–1.45, *p* = 0.138; PRa 0.94, 95% CI 0.84–1.06, *p* = 0.332; PRa 0.99, 95% CI 0.87–1.15, *p* = 0.976 and PRa 1.05, 95% CI 0.96–1.17, *p* = 0.269, respectively) (Table 4).

**Table 4:** Association between reports of physical violence inflicted by a partner in the last 12 months and internal migration flows. Peru ENDES 2015–2017 (n = 63,859)

Report of violence in the last 12 months	Crude model*			Adjusted model**		*P*
	PRc	95% CI		PRa	95% CI	
*Internal migration flows*						
Nonmigrant	1			1		
Recent migrant	1.19	1.08–1.31	0.001	1.05	0.96–1.17	0.269
Urban-urban	0.95	0.82–1.09	0.493	0.99	0.87–1.15	0.976
Urban–rural	1.34	1.07–1.66	0.008	1.18	0.95–1.45	0.138
Rural–urban	1.21	1.08–1.35	0.001	1.15	1.03–1.29	0.015
Rural-rural	1.04	0.93–1.17	0.414	0.94	0.84–1.06	0.332
*Age*						
30–49 years	1			1		
15–29 years	1.38	1.28–1.48	< 0.001	1.47	1.35–1.60	< 0.001
*Education*						
None/early education	1			1		
Primary	1.21	0.99–1.49	0.069	1.14	0.93–1.40	0.215
Secondary	1.27	1.04–1.55	0.019	1.15	0.93–1.42	0.201
Higher education (nonuniversity)	0.95	0.84–1.16	0.533	0.95	0.75–1.19	0.646
Higher education (university and postgraduate)	0.75	0.59–0.95	0.017	0.85	0.66–1.09	0.204
*History of physical aggression by father toward mother*						
No	1			1		
Yes	1.78	1.64–1.93	< 0.001	1.69	1.57–1.84	< 0.001
Does not know/Did not answer	1.41	1.11–1.81	0.005	1.33	1.03–1.70	0.027
*Native language*						
Spanish	1			1		
Quechua	1.16	1.03–1.30	0.011	1.14	1.01–1.28	0.038
Aymara	1.39	0.94–2.04	0.096	1.39	0.95–2.03	0.091
Other indigenous language/foreign language	1.02	0.65–1.59	0.947	0.97	0.61–1.54	0.899
*Paid occupation*						
No	1			1		
Yes	1.19	1.09–1.30	< 0.001	1.25	1.14–1.36	< 0.001
*Number of children*						
None	1			1		
One	1.32	1.05–1.66	0.018	1.27	1.01–1.61	0.042
Two	1.18	0.95–1.49	0.129	1.29	1.03–1.62	0.029
Three or more	1.40	1.12–1.76	0.003	1.59	1.25–2.01	< 0.001
*Marital/conjugal status*						
Married	1			1		
Cohabitating	1.63	1.47–1.79	< 0.001	1.40	1.27–1.56	< 0.001
Separated/Divorced/Widowed	1.73	1.52–1.97	< 0.001	1.49	1.28–1.75	< 0.001
*Another sexual partner in the last 12 months in addition to a stable partner*	1			1		
Yes	1.25	1.09–1.44	0.002	1.05	0.88–1.27	0.576
*Alcohol consumption by the partner in the last 12 months*						
No	1			1		
Yes	1.73	1.53–1.96	< 0.001	1.58	1.40–1.78	< 0.001
*Interviewee's duration of living in the place to which she migrated*						
Nonmigrant	1					
Less than 5 years	1.19	1.07–1.31	0.001			
5 years or more	1.11	1.02–1.22	0.021			
*Socioeconomic status*						
Lower quintile	1					
Second quintile	1.21	1.10–1.33	< 0.001			
Third quintile	1.14	1.02–1.27	0.019			
Fourth quintile	0.91	0.80–1.03	0.336			
Upper quintile	0.52	0.44–0.61	< 0.001			

PR: Prevalence ratio (c = crude, a = adjusted), 95 CI%: 95% confidence interval

*Crude generalized linear model of the logarithmic Poisson link log family. The results are presented asf prevalence ratios (PRc)

**Adjusted generalized linear model of the logarithmic Poisson link log family. The results are presented as prevalence ratios (PRa). For the entire analysis, complex sampling (svy) was considered, in addition to the model variables age, education level, occupation, number of children, history of physical aggression of the father toward the mother, marital status and frequency of alcohol consumption by the partner. Complex sampling (svy) is considered for the entire analysis

## Discussion

This is the first research to analyze internal migration patterns and IPV in Peru using a large population-based dataset. This study confirmed that there is a greater probability of IPV in the last 12 months among women who migrated from a rural area to an urban area than among those who did not migrate. In addition to this finding, the fact that rural–urban migration also accounts for the most common internal migration flow, involving two out of every ten women in the country, as well as more than four out of every ten women who migrated within the country, turns rural–urban migration into a factor associated with IPV that should be included in public health strategies in the Peruvian context. The results of the comparisons of IPV among women with different internal migration flows and nonmigrants complement the information from previous studies, including a study conducted in Peru with rural–urban migrant women that identified a high prevalence of domestic violence and related it with mental health problems [32]. In addition, research on married working couples who migrated from a rural area to an urban area in China found a high prevalence of IPV [15]. Finally, a review of the literature on rural–urban migrants from China found an association between health problems and migration that was not found for nonmigrants. Rural–urban migrants showed a higher prevalence of communicable diseases, women's health issues and noncommunicable diseases, all of which were related to work and risk behaviors and did not include an analysis of domestic violence (IPV) as a problem related to public health [33].

The mechanism through which rural–urban migration is associated with IPV could be the experiences of migrant women and their establishment in a new place, including experiences related to housing, health access and work. As a consequence of this new experience, the family dynamics become more complex according to the gender relations of the couple; it influences whether their socioeconomic status improves and means that one or both must engage in paid work and share the responsibility for childcare and domestic tasks [11]. In this new scenario, women face barriers to accessing care services for victims of family violence; these barriers include language barriers, ignorance of their rights and of the available services in general, fear of authorities, social isolation, family disintegration and shame [34]. In the absence of clear regulations aimed at rural–urban migrants, it is difficult for these women to adapt to health coverage systems, and they lack support for accessing new job opportunities, among other difficulties [35]. These barriers are also intertwined with the rural cultural context from which the women migrated, with values and cultural factors characteristics of collectivism, considering that since their birth, women belonged to a strong and cohesive group, probably an extended family, and when they move to the urban area, far from their family, they face particularly challenging situations, and not having family nearby to rely on, makes them more vulnerable to IPV [36].

In a study carried out in Peru for the 2007–2009 period, a difference in the prevalence of IPV according to the area of residence was reported, namely, living in an urban environment outside the coast and the mountains is associated with an increased probability of IPV compared with living in metropolitan Lima [37]. However, it is currently not enough to statistically consider these characteristics of the area of residence because nonmigrants and women in different migration flows have different risk factors, including the impact of climate change and its relationship with rural–urban flow, which is strongly predicted by agricultural employment and education level [38].

In our study, 42.9% of women reported having witnessed physical violence inflicted by their father on their mother during childhood. Although it is unknown whether this violence also took place during the prenatal or childhood stage, there is evidence that childhood, gestational exposure and even exposure during pregnancy to psychological stressors, including IPV, leads to increased methylation of the human GR promoter, which influences psychological function. This mechanism can be explained by the transgenerational epigenetic effect of stress and aggression on human behavior; in other words, exposure to violence early in life can have mental health consequences such as depression, abuse of psychoactive substances and increased vulnerability to IPV at a later stage in life [39–41].

This research included a large sample of women from a population-based study conducted in Peru that collected information annually between 2015–2017, which allowed the identification of internal migration profiles as well as various geographic and sociodemographic characteristics of the women according to whether they had experienced IPV in the last 12 months using a validated and internationally comparable scale [21].

However, this study is not without limitations. It was a cross-sectional study that did not consider the conditions of the population prior to migration, especially with respect to IPV, its frequency and intensity and the information reported by the couple. There is also no information on whether the women had migrated alone or the areas where they resided between childhood and the time of their interview. Although several potential confounding variables were included in the original study, others were not, such as history of abuse or trauma during childhood, the women’s relationship and dynamic with their partner, partners employment, whether they lived with or separate from the partner. Information about the women’s history of alcohol abuse, changes in socioeconomic status, the norms of the context and cultural practices that influence a couple’s formation and relationship and insertion into social support networks in their current setting is also not available in the ENDES. The sample sizes of some categories of variables, such as urban–rural migration and mother language, were small; therefore, the results should be interpreted with care. However, the results of this study with respect to rural–urban migration, which is the most commonly reported migration pattern, are important. Memory bias is likely due to the inclusion of self-reported retrospective information, such as the most frequent place of residence before 12 years of age and duration of residence in the current place and the history of physical aggression by the father toward the mother; additionally, there is a risk of social desirability bias when answering questions concerning violence.

## Conclusions

In Peru a developing country, rural–urban migration among women of childbearing age is a factor associated with a higher probability of IPV in the last 12 months.

The identification of women with this rural–urban migration pattern could help prioritize those that may experience a greater probability of physical and/or sexual violence, and along with other findings of this study, highlight the need to develop multisectorial and multidisciplinary strategies focused on rural–urban migrant women, with broad outreach and development of specific health, education, legal and labor services, including ensuring the safety of women and their children, improving access to physical and mental health services, health education, employment promotion services and legal counseling. It is necessary to develop longitudinal studies and studies in other countries and contexts that contribute to the development of deeper knowledge of the conditions of this association and public health strategies for the management and prevention of IPV. Studies should also be developed to identify what types of outreach and services would be most accessible and appropriate to the needs, priorities, and support of rural–urban migrants.

## Data Availability

This study was a secondary analysis of publicly available data of the Demographic Health Survey by the National Statistics institute of Peru can be accessed from http://iinei.inei.gob.pe/microdatos/.
